# Predicting Early Loss of Lateral Spread Response before Decompression in Hemifacial Spasm Surgery

**DOI:** 10.3390/life12010040

**Published:** 2021-12-27

**Authors:** Ryan Wing-Yuk Chan, Yung-Hsiao Chiang, Yi-Yu Chen, Yi-Chen Chen, Jiann-Her Lin, Yi-Syue Tsou

**Affiliations:** 1 Department of Neurosurgery, Taipei Medical University Hospital, Taipei 11031, Taiwan; b101095151@tmu.edu.tw (R.W.-Y.C.); ychiang@tmu.edu.tw (Y.-H.C.); 986065@h.tmu.edu.tw (Y.-Y.C.); 866288@h.tmu.edu.tw (Y.-C.C.); jiannher@me.com (J.-H.L.); 2 Taipei Neuroscience Institute, Taipei Medical University, Taipei 11031, Taiwan; 3 Department of Surgery, School of Medicine, Taipei Medical University, Taipei 11031, Taiwan

**Keywords:** hemifacial spasm (HFS), intraoperative monitoring (IOM), lateral spread response (LSR)

## Abstract

Recent studies have shown the evocation of lateral spread response (LSR) due to the compression of the facial nerve in hemifacial spasm (HFS). Intraoperative monitoring (IOM) of LSR could help locate neurovascular conflicts and confirm adequate micro-vascular decompression (MVD) while treatment of hemifacial spasm (HFS). However, studies on early LSR loss before decompression in HFS surgery are sparse, indicating the need to understand various perceptions on it. Therefore, we retrospectively analyzed 50 adult HFS patients who underwent MVD during the period of September 2018–June 2021. We employed IOM combining traditional LSR (tLSR) and dual LSR (dLSR). One patient was excluded owing to the lack of LSR induction throughout the surgery, while 49 were divided into groups A (*n* = 14) and B (*n* = 35), designated as with or without early LSR loss groups, respectively, and offending vessels were analyzed. The mean age of group A patients was significantly younger (47.8 ± 8.6) than that of group B (53.9 ± 10.6) (*p* = 0.0393). The significant predominating offending vessel in group A was the anterior inferior cerebellar artery (AICA, 78.57%). However, group B included those with AICA (28.57%), posterior inferior cerebellar artery (PICA, 22.86%), vertebral artery (VA) involved (25.71%), and combined AICA and PICA (22.86%). Group B exhibited poorer clinical outcomes with more complications. Conclusively, early LSR loss might occur in the younger population, possibly due to the AICA offending vessel. The compression severity of offending vessels may determine the occurrence of early LSR loss.

## 1. Introduction

Hemifacial spasm (HFS) is a disorder characterized by the intermittent, involuntary, tonic, or clonic twitching of facial muscles of unilateral face innervated by the ipsilateral facial nerves (cranial nerve [CN] VII) [1]. To date, microvascular decompression (MVD) is an optimal treatment for HFS, with a nearly 90% cure rate [2]. Under electromyography, HFS is represented as lateral spread response (LSR) of facial muscle to the stimulation of CN VII [3]. Specifically, the LSR in HFS is ascribed to the ephaptic transmission of neural impulses between different branches of the facial nerve [4]. Ideally, LSR loss can occur immediately while offending vessels were moved away from the facial nerve. However, early LSR loss before decompression during HFS surgery occurs frequently and remains unpreventable.

During MVD, intraoperative monitoring (IOM) is implemented to optimize surgical outcomes. In the event of early LSR loss, the guiding role of IOM is lost, and surgeons must rely solely on their experience to complete the operation. Therefore, this phenomenon requires exploration, which has been done only by a few studies [5,6,7,8,9,10,11,12]. Although the IOM of traditional LSR (tLSR) is very important, it is associated with many drawbacks including the absence of typical intraoperative waves from the initiation of MVD to completion, presence of the typical waves even after MVD, and unstable and surgical interference susceptible intraoperative waveforms [13].

However, the dual LSR (dLSR) [14] has better sensitivity and reliability than tLSR monitoring. Therefore, in this study, we explored the cause of early LSR loss in adult HFS patients who underwent MVD through combined tLSR and dLSR approaches. Specifically, the clinical outcomes were determined in terms of the comparative number of operations, offending vessels including anterior inferior cerebellar artery (AICA), posterior inferior cerebellar artery (PICA), and vertebral artery involved (VA), the indentation of the facial nerve.

## 2. Materials and Methods

**Figure 1:**
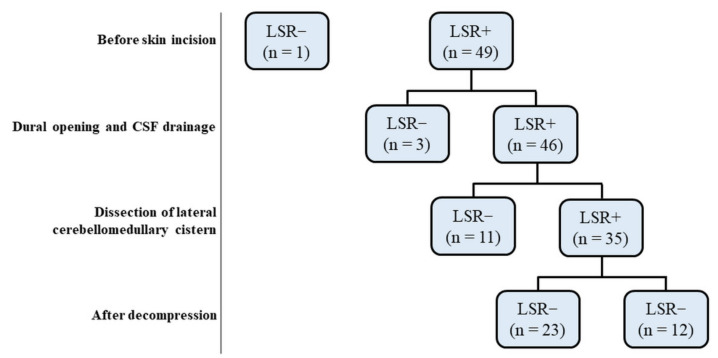
Flow diagram depicting the clinical evolution over time of the 50 patients with HFS treated by MVD. Groups were defined according to the presence or absence of LSR before and after decompression during MVD. HFS: Hemifacial spasm, LSR: Lateral spread response, MVD: microvascular decompression, CSF: cerebrospinal fluid.

Between 7 September 2018 and 30 June 2021, a total of 50 adult HFS patients treated by MVD were retrospectively analyzed (Figure 1). IOM using combined tLSR and dLSR measurement was used throughout the procedure. The protocols of dLSR were followed from a previous study by Zhang et al. [14].

### 2.1. Inclusion and Inclusion Criteria

The inclusion criteria were the diagnosis of HFS-based on the clinical presentation (such as involuntary eye and facial muscle contractions and eyelid closure) and identification of the contributing offending vessel through brain magnetic resonance imaging (MRI). The exclusion criteria were (1) the presence of other forms of facial movement disorders, (e.g., facial tics and facial dystonia); (2) symptomatic HFS secondary to posterior cranial fossa lesions, Chiari malformations, and aneurysms, etc.; and (3) Botox injection for spasm within 1 year before surgery; (4) patients with a history of craniotomy, and gamma-knife therapy; and (5) incomplete clinical data.

### 2.2. Surgery

All surgeries were performed at the same institute by a single surgeon, Yung-Hsiao Chiang, via a suboccipital retrosigmoid approach in the lateral decubitus position. After durotomy, cerebrospinal fluid (CSF) was drained sufficiently. The lateral cerebellomedullary cistern was dissected, and the compressed facial nerve was exposed through retracting the cerebellum. The offending vessels were identified in all patients, and several pieces of Teflon sponge were interposed between the offending vessel and the facial nerve’s root exit zone (REZ). The dura and later the skin was closed layer-by-layer after confirming no further existence of compression along the facial nerve.

### 2.3. Intraoperative Monitoring (IOM)

General anesthesia was performed on all the patients using propofol combined with sevoflurane (approximately 0.5 minimum alveolar concentration). Thereafter, the IOM using the combined traditional LSR (tLSR) and dual LSR (dLSR) was conducted throughout each surgical procedure. A commercial intraoperative neurophysiological monitoring system (Cadwell Cascade Elite, Kennewick, WA, USA) was used to amplify, filter, and display signals.

#### 2.3.1. tLSR Measurement

tLSR measurement was executed by recording the electric stimulation of the zygomatic branch of the facial nerve. For recording, 2 paired needle electrodes were placed in the orbicularis oris and mentalis muscles. The LSR was recorded using 50 summations by employing amplifiers with a frequency band of 20 Hz to 3 kHz. Electrical stimulation (0.2 milliseconds, rectangular wave, 3 Hz) was adjusted to supramaximal strength. The tLSR was continuously recorded and printed out at 3-min intervals.

#### 2.3.2. dLSR Measurement

The dLSR measurement was performed through the electric stimulation of the zygomatic and marginal mandibular branches of the facial nerve. The corresponding LSRs were recorded from the mentalis and orbicularis oculi by using amplifiers with frequency bands of 30 Hz to 5 kHz. If the waves of all four recording electrodes of the combined tLSR and dLSR disappeared completely or the amplitude decreased to less than 50% of the baseline level, the LSR was considered to be lost. If the waves of any of the recording electrodes of the combined tLSR and dLSR appeared, we considered the LSR to have existed.

### 2.4. Outcome Evaluation

All patients were regularly followed-up for a period ranging from 4 to 33 months and the surgical outcomes and complications were recorded. In particular, the satisfactory improvement status was defined in the terms of postoperative spasm resolution of ≥80% and patient feedback.

### 2.5. Statistical Analysis

Categorical variables (Gender, side of spasm, offending vessel, number of operations, indentation of facial nerve, and surgical outcome) were compared using cross tabs with a chi-squared test or Fisher’s exact test. Continuous variables (age and duration of symptom) were compared using the Mann-Whitney test. Statistical significance was defined at *p* < 0.05.

## 3. Results

**Figure 2:**
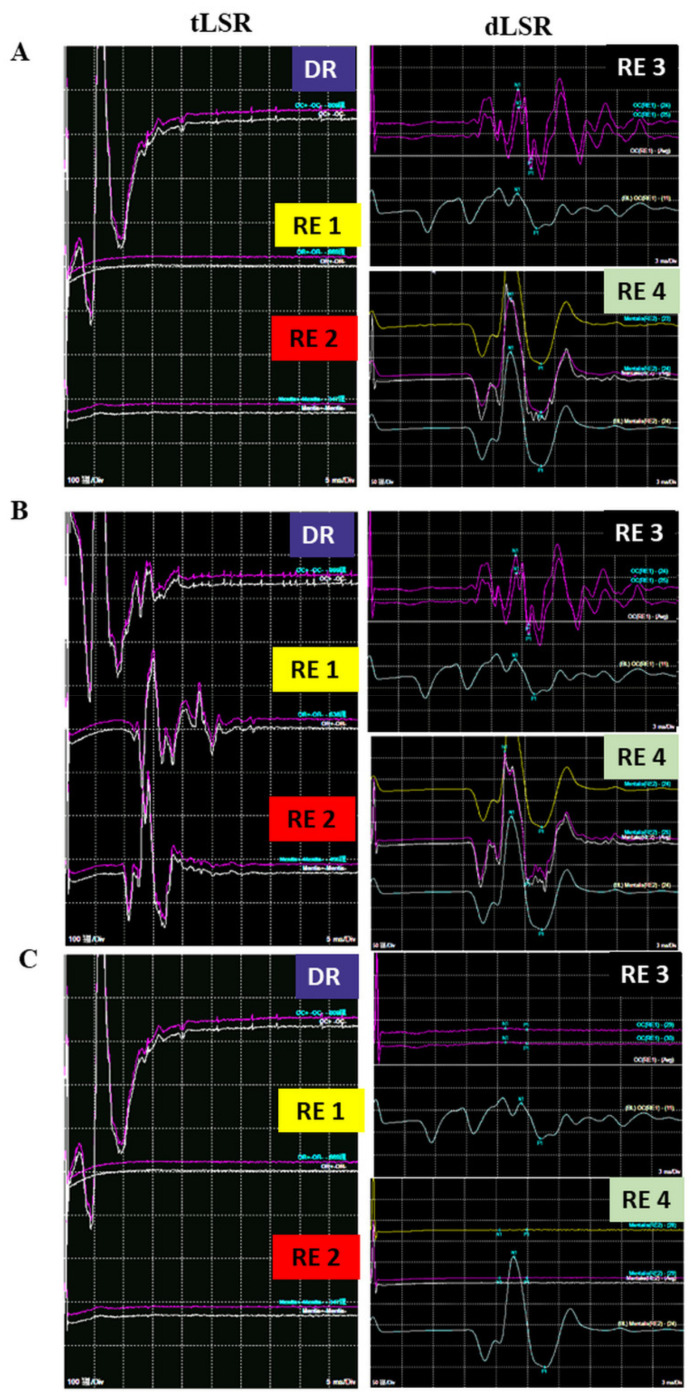
Representative image of early loss of LSR before decompression in an HFS patient. (**A**) before dural opening, the tLSR recorded from the orbicularis oris (RE1) and mentalis (RE2) are absent, but dLSR recorded from orbicularis oculi (RE3) and mentalis (RE4) existed. (**B**) After dural opening the tLSR, recorded from RE1 and RE2 reappeared, while dLSR recorded from RE3 and RE4 still existed. (**C**) After arachnoid dissection, the waves of all four recording electrodes disappeared and no longer seen. tLSR: traditional lateral spread response, dLSR: dual lateral spread response, HFS: Hemifacial spasm, DR: Direct recording; RE: Recording electrode; MVD: Microvascular decompression.

Following the IOM of 50 patients (Figure 1), one was excluded from the study owing to the lack of LSR induction throughout the procedure. The other 49 patients were divided into two groups i.e., A (*n* = 14) and B (*n* = 35), designated as with or without early LSR loss groups, respectively before decompression (Table 1). We found the mean age of group A significantly much younger than group B (*p* = 0.0393). The early LSR loss rate before decompression was 28.6%. In group A patients, LSR disappeared while CSF egressed after dural opening (*n* = 3) as well as during the dissection of the lateral cerebellomedullary cistern (*n* = 11) (Figure 2).

Group B patients were observed with persisted LSR (*n* = 12) as well as disappeared LSR (*n* = 23) after decompression. 

**Figure 3:**
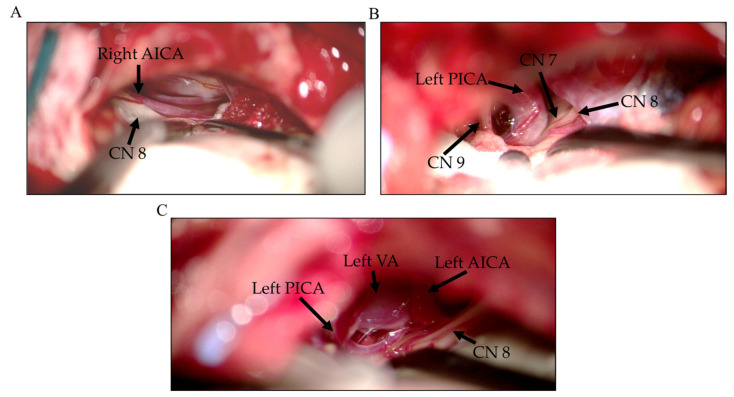
Representative intraoperative images of offending vessels during MVD surgery in HFS patients. (**A**) AICA, (**B**) PICA, and (**C**) the vertebral artery (VA) with AICA and PICA. MVD: microvascular decompression, HFS: Hemifacial spasm. AICA: Anterior inferior cerebellar artery; PICA: Posterior inferior cerebellar artery, CN: Cranial nerve.

Of the patients in group A, three (21.4%) were male and seven (50%) had left-sided symptoms with a mean age of 47.8 ± 8.6 (29~62) years, and the mean duration before surgery was 5.7 ± 3.0 (1~10) years. Notably, none of the 14 patients received surgery due to recurrent HFS. The offending vessels (Figure 3) identified in these patients include the AICA (*n* = 11, 78.6%), PICA (*n* = 1, 7.1%), VA-involved (*n* = 1, 7.1%), and combined AICA and PICA (*n* = 1, 7.1%). One facial nerve indentation was identified in group A. Of the group B patients, 18 (51.4%) were men, and 21 had left-sided symptoms with the mean age being 53.9 ± 10.6 (33–75) years and the mean duration before surgery being 6.2 ± 4.4 (1–22) years. Moreover, the 30 patients underwent the first operation, while 5 patients received surgery due to recurrent HFS. The offending vessels identified in these patients were as follows: the AICA (*n* = 10, 28.57%), PICA (*n* = 8, 22.86%), VA-involved (*n* = 9, 25.71%), and combined AICA and PICA (*n* = 8, 22.86%). Two facial nerve indentations were identified in group B.

Following surgery, the immediate cure rates in groups A and B were 85.7% (*n* = 12) and 68.6% (*n* = 24), respectively (Table 2). Further, a delayed resolution (7 days post-surgery was experienced in group A (*n* = 2) as well as group B (*n* = 5). In the long-term (1 year), the cure rate was increased to 100% (*n* = 14) and 82.9% (*n* = 29) in group A and B, respectively. (Table 2). Eventually, six patients’ symptoms were not ameliorated. Although the difference remains statistically insignificant, it seems that compared to group B, the group A has a higher probability of achieving favorable outcomes.

As shown in Table 3, no complications were encountered in group A, while group B revealed the cases of delayed facial palsy (*n* = 2), tinnitus (*n* = 2), abducens palsy (*n* = 2), and acute subdural hematoma (*n* = 1).

Notably, except for the two patients with tinnitus, all patients were fully recovered from their complications. No cerebellar hemorrhage, infarction, CSF leakage, infection, or mortality occurred in either group. Taken together, our results showed a younger mean age of group A compared to group B. The components of the offending vessels differed significantly with AICA as a predominant offending vessel in group A. However, considerably more VA-involved and PICA were identified as offending vessels in group B. The postoperative clinical outcomes also differed between the groups with the favorable outcomes in predominantly in group A.

## 4. Discussion

IOM can help surgeons in identifying the precise location of neurovascular conflict, ensuring adequate decompression [5], and ascertaining the recurrence of HFS. However, the contribution of IOM during decompression surgery for the treatment of HFS remains to be clearly understood. Notably, a recent study highlighted the etiology of unsuccessful first time MVD in HFS patients that broadly differs (50–98%) and could be attributed to the omission of offending vessels [15]. Therefore, the MVD procedure can be improved through IOM which could not only identify the specific offending vessel among the multiple ones but also lower the complication rate of MVD for HFS [16]. Similarly, a few previous studies have also supported the usefulness of IOM in MVD [5,7,10,16,17,18,19,20] by decreasing the learning curve and providing intraoperative guidance to obtain the best results [5]. According to Sekula et al., the chance of cure is 4.2 times greater if LSR eliminates during surgery compared to persisted state [17]. However, some researchers have propounded that IOM may not be a reliable indicator of long-term postoperative outcomes of HFS surgery [11,21,22,23,24,25]. In a prospective study, Wei et al. showed no significant difference in clinical outcomes between two groups undergoing MVD with and without IOM [11]. A systematic review by Nugroho et al. demonstrated that LSR disappearance may be an independent predictor of short-term outcomes but not a reliable predictor of long-term outcomes [26]. These studies have been conducted under the premise that LSR could be evoked initially and correctly reflected the situation of facial nerve being compressed. However, early LSR loss before decompression is frequently encountered. Under conditions involving early LSR loss, the adequate decompression under electrophysiological confirmation is not possible. Therefore, surgeons are required to complete the surgery without the help of LSR monitoring.

Early LSR loss before decompression may occur during the CSF drainage or arachnoid membrane dissection [9]. The frequency of premature LSR abolition ranged from 6.8% to 34% in various studies [5,6,7,8,10,11,12]. As per previous studies, the prognosis of early LSR loss before decompression in HFS surgery varies with the corresponding long-term (at least 1 year) cure rate between 60% and 100% [5,6,7,8]. A few studies have discussed the mechanism underlying early LSR loss before decompression in HFS surgery [8,10,12]. Cerebrospinal fluid drainage, flocculus retraction, and arachnoid tissue dissection could temporarily cause an anatomical shift in the neurovascular relationship, resulting in the transient decompression of the facial nerve. Mooij et al. considered the role of LSR as “indirect confirming”, while LSR disappeared after cerebrospinal fluid drainage [5]. Therefore, the interposition of the Teflon felt pledget is required to permanently maintain the decompression. Furthermore, continuous LSR monitoring throughout surgery is suggested to prevent LSR reappearance. Early LSR loss also signifies that the compression force applied by the offending vessels is relatively small and may be easily affected by a subtle environmental change, such as CSF egress. This may explain why the components of the offending vessels differed between groups A and B. The VA as an offending vessel has a large diameter and is stiff; hence it difficult to be mobilized and compress indirectly through the underlying vessel to the REZ in tandem fashion. Therefore, the compression force applied by the VA is larger than by the other offending vessels. The PICA may also act as offending vessel since its proximal region circumvent toward VII and VIII CN before descending to pass between IX and XI CNs. The arterial loop seems to compress the REZ more severely than the AICA, which often contacts the facial nerve without severe compression (Figure 3). Therefore, PICA transposition and re-transposition are often required during decompression in MVD [27]. Furthermore, Jiang et al. reported that the components of offending vessels vary between patients with or without early LSR loss [8]. In their study, no VA-involved vessel was identified as an offending vessel in the group of patients with early LSR loss. Whereas, in the group without early LSR loss, 30 patients had VA-involved vessels. Moreover, the number of facial nerve indentations varied significantly between the two groups (0 vs. 39) *(p =* 0.0355), implying that the compression force applied by the offending vessel to the REZ is the key factor in predicting the occurrence of early LSR loss in HFS surgery.

The reason for younger patients and preponderance of AICA in group A is probably due to rapidly progressive symptoms as suggested by Park et al. [28]. In addition, they proposed that mild vascular compression and the consequent absence of nerve indentation rather than severe vascular compression and consequent nerve indentation give rise to rapid HFS progression. The long-term prognosis for group A seemed to be more favorable than group B. This may be due to the greater number of complex offending vessels in group B. Moving complex offending vessels away from the REZ requires more precise techniques and greater effort, increasing the surgical difficulty. Our results are in line with a report showing young-onset HFS with their clinical presentation similar to the elderly [29]. However, our result contradicts the findings of Kim et al. [6], who revealed that patients with early LSR loss had poorer outcomes than those with late LSR disappearance. Notwithstanding, patients with persistent LSR were not included in this study.

Numerous factors may contribute to the higher number of complications in group B. The degree of cerebellar retraction required for the decompression and visualization of the REZ compressed by the complex offending vessels was greater. Furthermore, 12 patients in group B with persistent LSR after decompression may have an increased risk of complications due to the repeated exploration of the facial nerve. Some of these patients, the exact offending vessels may have been either missed or misidentified. In addition, age may be a predictor of surgical outcome. Engh et al. proposed that perhaps older patients with failed MVD are more likely to have chronic, irreversible neuropathy of the facial nerve than younger patients [30]. To note, the tinnitus was not observed before surgery. It developed after surgery, therefore has been considered a postoperative complication. However, the subdural hematoma might be associated with excessive cerebrospinal fluid (CSF) aspiration. Hence, we suggest avoiding and compensating for the CSF loss with artificial cerebrospinal fluid adequately to avoid subdural hematomas after microvascular decompression. Apart from various significant outcomes, the limitations in the current study included a single-center study and small sample size and, hence, the absence of the multivariate analysis of subgroups and short follow-up period. Therefore, future studies with a large sample size and long-term observations are needed to corroborate our findings.

## 5. Conclusions

Our results indicated that early LSR loss may occur in younger HFS patients. This might be predominantly attributed to AICA as the offending vessel. The occurrence of early LSR loss ahead of decompression may be attributed to less severity of compression applied by offending vessels. Continuous IOM throughout MVD surgery is still suggested to ensure no recurrence of LSR.

## Figures and Tables

**Table 1:** Demographics and clinical characteristics of the study population. Group A: Early LSR loss, Group B: Non-early LSR loss. AICA: Anterior inferior cerebellar artery; PICA: Posterior inferior cerebellar artery; VA: Vertebral artery.

	Group A (*n* = 14)	Group B (*n* = 35)	*p*-Value
Gender (M/F)	3/11	18/17	0.0552
Side (L/R)	7/7	21/14	0.5228
Mean age (years)	47.8 ± 8.6	53.9 ± 10.6	0.0393
Mean duration (years) (mean ± SD)	5.7 ± 3.0	6.2 ± 4.4	0.9911
No. of operation (1st/2nd)	14/0	30/5	0.3032
Offending vessels			
AICA	11 (78.57%)	10 (28.57%)	0.0205
PICA	1 (7.14%)	8 (22.86%)	
VA involved	1 (7.14%)	9 (25.71%)	
AICA + PICA	1 (7.14%)	8 (22.86%)	
Indentation of the facial nerve	1	2	1

**Table 2:** Surgical outcomes of two groups. Group A: Early LSR loss, Group B: Non-early LSR loss.

	Post-Operative		Long-Term	
	Group A	Group B	Group A	Group B
Cured	12	24	14	29
Uncured	2	11	0	6
*p*-value	0.2969		0.1639	

**Table 3:** Complications of two groups. Group A: Early LSR loss, Group B: Non-early LSR loss.

	Complications	
	Group A	Group B
Delayed facial palsy	0	2
Tinnitus	0	2
Abducens palsy	0	2
Subdural hematoma	0	1

## Data Availability

The data presented in this study are available on request from the corresponding author.
